# Can computer‐assisted implant surgery improve clinical outcomes and reduce the frequency and intensity of complications in implant dentistry? A critical review

**DOI:** 10.1111/prd.12458

**Published:** 2022-08-04

**Authors:** Atiphan Pimkhaokham, Sirimanas Jiaranuchart, Boosana Kaboosaya, Sirida Arunjaroensuk, Keskanya Subbalekha, Nikos Mattheos

**Affiliations:** ^1^ Department of Oral and Maxillofacial Surgery, Faculty of Dentistry Chulalongkorn University Bangkok Thailand; ^2^ Department of Dental Medicine Karolinska Institute Stockholm Sweden

**Keywords:** clinical outcomes, computer‐assisted implant surgery, oral health‐related quality of life, patient‐reported outcomes

## Abstract

Computer‐assisted implant surgery (CAIS), either static or dynamic, is well documented to significantly improve the accuracy of implant placement. Whether the increased accuracy leads to a corresponding improvement in clinical outcomes has not yet been systematically investigated. The aim of this critical review was to investigate whether the use of CAIS can lead to reduction of complications as well as improved clinical and patient‐reported outcomes (PROs) when compared with conventional freehand implant surgery. A comprehensive online search was conducted to identify studies where implants were installed with static computer‐assisted implant surgery (s‐CAIS)or dynamic computer‐assisted implant surgery(d‐CAIS) or combinations of the two, either compared with conventional free‐hand implant placement or not. Seventy‐seven studies were finally included in qualitative analysis, while data from three studies assessing postsurgical pain were suitable for a meta‐analysis. Only a small number of the available studies were comparative. The current evidence does not suggest any difference with regard to intraoperative complications, immediate postsurgical healing, osseointegration success, and survival of implants placed with CAIS or freehand protocols. Intraoperative and early healing events as reported by patients in randomized clinical trials (RCTs) did not differ significantly between CAIS used with flap elevation and conventional implant placement. There is limited evidence that increased accuracy of placement with CAIS is correlated with superior esthetic outcomes. Use of CAIS does not significantly reduce the length of surgeries in cases of single implants and partially edentulous patients, although there appears to be a more favorable impact in fully edentulous patients. Although CAIS alone does not seem to improve healing and the clinical and PRO, to the extent that it can increase the utilization of flapless surgery and predictability of immediacy protocols, its use may indirectly lead to substantial improvements in all of the above parameters.

## 1 INTRODUCTION

Computer‐assisted implant surgery (CAIS) includes two major technological pathways, typically distinguished as static and dynamic Computer‐assisted implant surgery (d‐CAIS) or real‐time navigation. Static Computer‐assisted implant surgery (S‐CAIS) technology utilizes a surgical guide for guiding the osteotomy and implant installation, while the d‐CAIS system guides the surgeon during osteotomy and implant placement through real‐time imaging by means of optical tracking devices. Both systems are currently widely used and have been well documented to help surgeons achieve significantly higher accuracy of implant placement than conventional freehand surgery. Several recent systematic reviews and meta‐analyses have shown superior outcomes in terms of accuracy for both static^1^ as well as d‐CAIS.^2^, ^3^

Accuracy of implant placement, however, is not the end purpose in itself. Instead, by offering superior accuracy, the endpoint of CAIS is to facilitate superior clinical outcomes, by reducing failures, complications, and adverse effects. There are many ways that increased accuracy is expected to influence clinical outcomes. Some authors have suggested that CAIS could reduce intraoperative complications by helping the surgeon avoid damaging sensitive anatomic structures such as the mandibular nerve and the sinus, as well as avoid proximity to roots of neighboring teeth.^4^ Some have suggested CAIS could reduce the invasiveness, complexity, and duration of surgical interventions, while others have reported increasing patients’ satisfaction and acceptance.^5^ Finally, as this technology aims to empower the proper prosthetic‐driven implant placement, it could potentially impact the long‐term outcomes of implant therapy by allowing implant placement in the optimal position and angulation for prosthetic designs that could promote sustainable esthetic outcomes and health of the peri‐implant tissue.^6^, ^7^ Optimal positioning of the implants may also reduce the cost of reconstruction, by allowing the use of stock abutments and minimizing the need for expensive customized solutions. Initial research on CAIS and consequent systematic reviews have primarily focused on reporting accuracy, although some authors acknowledge that survival rates, complications, patient‐centered outcomes, and socioeconomic benefits are essential variables that cannot be ignored.^2^ As these technologies are increasingly applied, assessing overall effectiveness and efficiency is critical to identify the potential and limitations of such systems, as well as help clinicians to make evidence‐based decisions.

### 1.1 Aims

The primary aims of this critical review were to investigate whether the use of CAIS can lead to:

1. Reduction of the frequency and extent of complications (intraoperative, postoperative, and medium/long term).
2. Improvement of patient‐reported outcomes (PRO) and/or patient‐reported experience (PRE).

The secondary aims were to report the influence of CAIS on:

3 Clinical outcomes related to complications (plaque index, bleeding on probing (BOP), probing depth, keratinized mucosa, marginal bone loss (MBL), esthetic outcomes, duration of surgery).
4 Clinical outcomes related to the overall efficiency of CAIS (duration of the surgery, experience, and training of the operator).

## 2 MATERIALS AND METHODS

### 2.1 Methodology

A comprehensive online search was conducted in PubMed aiming to identify clinical trials in the last 10 years, where implants were installed with static or dynamic CAIS or combinations of the two, either compared with conventional free‐hand implant placement or not. The electronic database PubMed was searched in February 2022 for articles in English with a limit of 10 years using the search query: {“Computer assisted implant surgery” OR “Computer aided implant Surgery” OR “guided implant Surgery” OR “implant navigation” OR “static guided” OR “dynamic guided”} AND Dental. In addition, a manual search was conducted on the reference lists of four recent systematic reviews and meta‐analyses.^1^, ^2^, ^3^

### 2.2 Qualitative data synthesis and quantitative/statistical analysis

**FIGURE 1:**
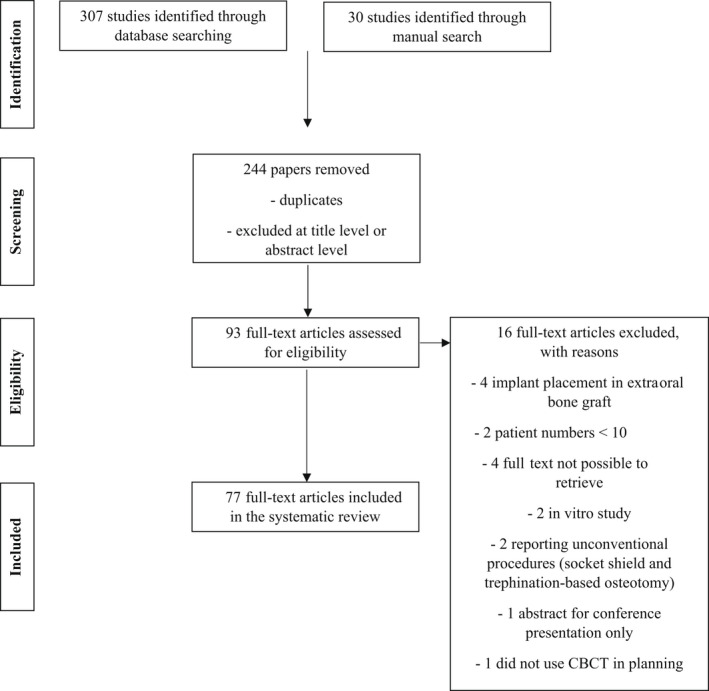
Flowchart of the articles included in the review. CBCT, cone beam computed tomography

In most cases, the heterogeneity of the data allowed only collective qualitative analysis. In the case of postsurgical PROs of pain originating from the first week of healing, a meta‐analysis was conducted on three studies.^8^, ^9^, ^43^ The meta‐analysis was conducted using the software RevMan version 5.4 (Review Manager, the Cochrane Collaboration, 2020). Mean and standard deviation (SD) of patient‐reporting pain scores at the designated time points derived from the selected articles were used as quantitative data, comparing the severity of pain between different types of surgery (static and d‐CAIS data compiled vs freehand placement (FH), s‐CAIS only vs FH). Thus, the standardized mean difference was applied to identify the magnitude of the effect and calculated 95% confidence interval. Then the random effect model was used for analysis. The heterogeneity across the studies was assessed by Chi‐squared and *I* ^2^ tests. Forest plots were constructed to represent the results of meta‐analysis of the included studies. A *P* value of less than .05 was judged to have statistical difference. Data were extracted and assessed by 77 studies (Table 1 and Figure 1).

**TABLE 1:** Overview of the studies analysed in the present review, including the study design, types of Computer Assisted Implant Surgery utilised and the studied outcomes

Author/year	Design	Type of surgery	Studied outcomes									
			Stability	Accuracy	PROMs	Success/survival	Complications	Workflow variations	Peri‐implant tissue	Esthetic	Duration	Other
Almahrous et al/2020	RCT	s‐CAIS, FH			•			•	•		•	
Aydemir et al/2020	RCT	d‐CAIS, FH		•			•					
Bencharit et al/2018	Cross‐sectional study	s‐CAIS		•				•				
Block et al/2016	Prospective study	d‐CAIS		•				•				
Block et al/2017	Prospective study	d‐CAIS, FH		•								•
Cassetta et al/2012	Retrospective	s‐CAIS		•				•				•
Cassetta et al/2013	Retrospective	s‐CAIS		•				•				
Cassetta et al/2014	Retrospective	s‐CAIS		•				•				•
Cassetta et al/2017	RCT	s‐CAIS		•								•
Cassetta et al/2020	Prospective cohort	s‐CAIS		•								•
Chai et al/2020	Pilot Clinical trial	s‐CAIS		•				•				
Cristache et al/2021	RCT	s‐CAIS			•	•		•			•	
D’Haese et al/2012	Prospective clinical trial	s‐CAIS	•	•		•	•					
De Souza et al/2022	Retrospective clinical study	s‐CAIS		•				•				
Deeb et al/2018	Retrospective case‐control	s‐CAIS							•			
Derksen et al/2019	Prospective cohort study	s‐CAIS		•		•	•					
Di Giacomo et al/2012	Prospective study	s‐CAIS		•		•	•					
Engkawong et al/2021	RCT	s‐CAIS, d‐CAIS, FH			•						•	
Fand et al/2019	Prospective clinical trial	s‐CAIS		•								
Fürhauser et al/2015	Retrospective study	s‐CAIS		•						•		
Geng et al/2015	Prospective clinical trial	s‐CAIS		•				•				
Kaewsiri et al/2019	RCT	s‐CAIS, d‐CAIS	•	•							•	
Kiatkroekkrai et al/2019	RCT	s‐CAIS		•				•				
Kivovics et al/2020	RCT	s‐CAIS		•								•
Ko et al/2021	RCT	s‐CAIS	•			•	•		•			
Kunavisarut et al/2021	RCT	s‐CAIS, FH			•	•						
Kuo et al/2021	Case series	d‐CAIS	•			•			•	•		
Lee et al/2013	Prospective clinical trial	s‐CAIS		•								
Lerner et al/2020	Retrospective study	s‐CAIS	•		•	•	•			•		
Lin et al/2020	Prospective clinical study	s‐CAIS		•			•					
Makarov et al/2021	Prospective pilot cohort study	s‐CAIS	•			•						
Mangano et al/2018	Prospective clinical study	s‐CAIS				•	•				•	
Matsumura et al/2021	Retrospective clinical study	s‐CAIS		•								
Meloni et al/2013	Prospective case series	s‐CAIS				•			•			
Meloni et al/2013	Prospective clinical study	s‐CAIS				•			•			
Mouhyi et al/2019	Retrospective study	s‐CAIS	•			•	•				•	
Naziri et al/2016	Prospective clinical study	s‐CAIS		•		•		•				
Ngamprasertkit et al/2021	Randomized clinical trial	s‐CAIS, FH		•								
Nocini et al/2013	Retrospective study	s‐CAIS	•			•	•					
Özden Yüce et al/2020	Prospective cohort study	s‐CAIS, FH		•	•						•	
Park et al/2020	RCT	s‐CAIS		•								
Peñarrocha et al/2012	Case control study	s‐CAIS, FH			•	•	•		•			
Pettersson et al/2012	Prospective cohort study	s‐CAIS		•								
Polizzi et al/2013	Retrospective study	s‐CAIS				•			•			
Pozzi et al/2021	Prospective cohort study	d‐CAIS	•			•			•			
Sancho‐Puchades et al/2019	RCT	s‐CAIS, FH			•	•					•	
Schelbert et al/2019	Prospective cohort study	s‐CAIS		•								
Schnutenhaus et al/2016	Retrospective study	s‐CAIS	•	•				•				
Schnutenhaus et al/2020	Prospective clinical study, RCT	s‐CAIS	•	•				•				
Skjerven et al/2019	Prospective cohort study	s‐CAIS		•		•						
Smitkarn et al/2019	RCT	s‐CAIS	•	•								
Søndergaard et al/2021	RCT	s‐CAIS, FH		•	•	•					•	•
Stefanelli et al/2019	Retrospective study	d‐CAIS		•								•
Sun et al/2020	Prospective cohort study	s‐CAIS, d‐CAIS, FH		•								
Van de Wiele et al/2015	Prospective study	s‐CAIS		•								•
Varga et al/2020	RCT	s‐CAIS, FH		•				•				
Velasco‐Ortega et al/2021	Prospective clinical study	s‐CAIS				•			•			
Velasco‐Ortega et al/2021	Prospective cohort study	s‐CAIS				•			•			
Vercruyssen et al/2014	RCT	s‐CAIS, d‐CAIS, FH		•								
Vercruyssen et al/2015	RCT	s‐CAIS, d‐CAIS, FH		•								
Vercruyssen et al/2016	RCT	s‐CAIS			•							
Verhamme et al/2015	Prospective study	s‐CAIS		•								
Vieira et al/2013	Prospective study	s‐CAIS	•	•								
Vinci et al/2020	Retrospective study	s‐CAIS				•						
Yimarj et al/2020	RCT	s‐CAIS, d‐CAIS		•								
Youk et al/2014	Cross‐Sectional Survey	s‐CAIS, FH			•							
Younes et al/2018	RCT	s‐CAIS, FH		•								
Younes et al/2019	RCT	s‐CAIS, FH		•							•	•
Zhao et al/2014	Prospective clinical study	s‐CAIS		•		•	•					
Zhu et al/2021	Retrospective study	s‐CAIS		•								

Abbreviations: d‐CAIS, dynamic computer‐assisted implant surgery; FH, freehand placement; PROM, patient‐reported outcome measure; RCT, randomized clinical trial; s‐CAIS, static computer‐assisted implant surgery.

### 2.3 Frequency/extent of complications and clinical outcomes with CAIS compared with conventional placement

#### 2.3.1 Intraoperative (surgical) complications

Intraoperative complications reported in the literature include the absence of implant stability,^10^, ^11^, ^12^, ^13^ surgical template fractures,^14^ instability/misfit of the surgical guide,^13^, ^15^, ^16^ bone dehiscence after implant placement,^14^, ^17^ and anatomic limitation/problematic access for s‐CAIS drills.^17^, ^18^ Of 10 studies reporting intraoperative complications (Table 2), only one compared outcomes between s‐CAIS and FH.

**TABLE 2:** Studies reporting surgical complications (intraoperative and postoperative)

Author/year	Design	Location	Edentulism	Type of surgery	No. of patients/implants	No. of implants/patient	Observation period	Surgical complications	
								Intra‐op.	postop.
Aydemir et al/2020	Split‐mouth RCT	PS	SI	d‐CAIS, FH	60/86	1.43	n/a	Deficient stability of the radiopaque stent during surgery in 2 patients (3.33%)	n/a
Derksen et al/2019	Prospective cohort study	PS	PE	s‐CAIS	66/145	2.19	24 mo	1. Impossible to use the long guide drill in 37 area for 1 implant (0.68%) 2. Buccal dehiscence, 2 implants (1.37%)	n/a
D’Haese et al/2012	Prospective clinical trial	AZ, PS	PE	s‐CAIS	13/78	6.00	12 mo	Insufficient primary stability, 3 implants (3.84%)	Abscess formation caused implant loss, 1 implant (1.28%)
Di Giacomo et al/2012	Prospective study	AZ, PS	PE, FE	s‐CAIS	12/62	5.16	30 mo	1. Lingual soft tissue was pulled by drill, 4 implants (6.45%) 2. Wider implant than planned were used to improve primary stability, 4 implants (6.45%) 3. Implant instability, 2 implants (3.22%)	1. Prolonged severe pain, in 1 patient (1.61%) 2. Gingival inflammation in 2 patients (3.22%)
Ko et al/2021	RCT	AZ, PS	SI, PE	s‐CAIS	72/187	2.59	12 mo	Deficient primary stability, 7 out of 93 implants in immediate loading group (7.53%)	n/a
Lerner et al/2020	Retrospective study	AZ, PS	PE, FE	s‐CAIS	12/110	9.16	12 mo	n/a	Peri‐implant mucosal inflammation with bleeding on probing after 3 mo in 2 implants (1.8%)
Lin et al/2020	Prospective clinical study	AZ, PS	SI, PE	s‐CAIS	21/43	2.04	n/a	Implant were inserted free‐handed due to the limited mouth opening, 7 implants (16.28%)	Implants were removed due to pain, 2 implants (4.65%) in 1 patient
Mangano et al/2018	Prospective clinical study	AZ, PS	SI, PE	s‐CAIS	19/36	1.89	12 mo	Inadequate fit and unsatisfactory stability, 1 of 28 stents (3.57%)	n/a
Mouhyi et al/2019	Retrospective study	AZ, PS	SI, PE	s‐CAIS	38/110	2.89	12 mo	1. Deficient primary stability in 2 implants (1.8%) 2. Inadequate fit and stability of stent, 2 stent (5%)	1. Pain and swelling, 2 patients (5.26%) 2. Peri‐implant mucositis, 2 implants (1.8%)
Nocini et al/2013	Retrospective study	AZ, PS	FE	s‐CAIS	65/342	5.26	12‐60 mo (mean 32.87 mo)	1. Fracture of stent, 1 stent (1.54%) 2. Implants deviated from the planned position with vestibular bone dehiscence, 2 implants (0.58%) 3. Implant loss due to fracture of maxilla, 5 implants (1.46%)	n/a
Peñarrocha et al/2012	Case control study	n/a	SI, PE	s‐CAIS FH	12/19 12/22	1.58 1.83	3 mo	Inadequate fit of surgical guide (11/24, 45.83%)	n/a
Zhao et al/2014	Prospective clinical study	AZ, PS	PE	s‐CAIS	11/31	2.81	n/a	n/a	Implant loss, 1 implant (3.22%) in a deep bite patient with immediate loading

Abbreviations: AZ, esthetic zone; d‐CAIS, dynamic computer‐assisted implant surgery; FE, fully edentulous; FH, freehand placement; n/a, the data were not provided in the articles; PE, partially edentulous; PS, posterior; SI, single implant; s‐CAIS, static computer‐assisted implant surgery; RCT, randomized clinical trial.

##### Primary stability and related outcomes

###### Presence/absence of clinical primary stability

Four studies reported outcomes related to the implant primary stability, none of which compared outcomes between CAIS and FH. One comparative RCT by Ko et al^12^ assessed clinical stability between immediate loading and delayed loading protocol with s‐CAIS and reported that seven out of 93 implants (7.53%) (five maxilla/two mandible) in four out of 36 patients had insufficient implant stability in the immediate loading group. In other prospective and retrospective studies, an absence of clinical stability after placement with s‐CAIS varied from 1.8% to 3.84%^10^, ^11^, ^13^ (Table 2).

###### Quantitative measures of primary stability

Eleven studies assessed or reported primary stability after implant placement utilizing diverse outcomes measures, including insertion torque value (ITV), reverse torque (RT), and resonance frequency analysis (RFA) (Table 3). Five of these studies were comparative, two RCTs comparing static computer‐assisted with freehand implant surgery and two RCTs comparing two different s‐CAIS protocols. Smitkarn et al,^19^ in an RCT, found statistically significantly higher implant stability quotient (ISQ) value in implants inserted freehand (bucco‐lingual 72 ± 9, mesio‐distal 72 ± 11) than with s‐CAIS (bucco‐lingual 63.5 ± 12, mesio‐distal 65 ± 12). The same study found statistically significantly better ITV in implants inserted by freehand (35 ± 11 Ncm) than with s‐CAIS (22.5 ± 20 Ncm). In the second RCT comparing freehand with s‐CAIS, Kaewsiri et al^20^ reported that the minimum of 25 Ncm insertion torque was reached in all cases in both groups. Two more RCTs assessed primary stability between different s‐CAIS techniques. Ko et al^12^ found a higher ISQ value in the delayed implant loading group (72.89 ± 7.85) compared with the immediate implant loading group (70.18 ± 14.45), although all implants reached the minimum of 25 Ncm insertion torque. Schnutenhaus et al^21^ compared mean ISQ values between implants placed with s‐CAIS and alveolar ridge preservation (63 ± 8.75) and nonaugmented sites (64.12 ± 7.88). Other prospective and retrospective studies with s‐CAIS have only reported that all implants displayed values above the clinically acceptable thresholds of ITV 25 Ncm, RT 20 Ncm, or ISQ of 55.^13^, ^14^, ^22^, ^23^, ^24^, ^25^ With regards to d‐CAIS, Pozzi et al,^24^ in a case series with 10 fully edentulous patients/60 implants, reported a mean ISQ of 71 ± 2.8.

**TABLE 3:** Studies included in the analysis for primary stability

Author/year	Design	Location	Edentulism	Type of surgery	No. of patients/implants	No. implants/patient	Observation period	Primary stability		
Kaewsiri et al 2019	RCT	AZ, PS	SI	s‐CAIS d‐CAIS	30/30 30/30	1 1	12 mo	ITV > 25 Ncm in all implants		
Ko et al 2021	RCT	AZ, PS	SI, PE	s‐CAIS	72/187	2.59	12 mo	Mean ± SD of ISQ: DL: 72.89 ± 7.85 IL: 70.18 ± 14.45		
Kuo et al 2022	Case Series	AZ	SI, PE	d‐CAIS	10/10	1.00	12 mo	ITV >25 Ncm in all implants		
Lerner et al 2020	Retrospective study	AZ, PS	PE, FE	s‐CAIS	12/110	9.16	12 mo	1. ITV <35 = 22.7% of implants, > 35 = 77.3% of implants 2. ISQ <55 = 25.5% of implants 55‐85 = 74.5% of implants		
Makarov et al 2021	Prospective pilot cohort study	AZ, PS	FE	s‐CAIS	10/55	5.50	12 mo	ITV > 35 Ncm in all implants		
Mouhyi et al 2019	Retrospective study	AZ, PS	SI, PE	s‐CAIS	38/110	2.89	12 mo	RT 20 Ncm (applying immediately after implant placement)		
Nocini et al 2013	Retrospective study	AZ, PS	FE	s‐CAIS	65/342	5.26	12‐60 mo (mean 32.87 mo)	1. median of ITV 40 Ncm, IQR 35‐50 2. ITV > 30 Ncm in all implants placed in smokers and in the mandible		
Pozzi et al 2021	Prospective cohort study	AZ, PS	PE, FE	d‐CAIS	10/60	6	14‐18 mo (mean ± SD 16.2 ± 1.7 mo)	Mean ± SD ISQ: 71 ± 2.8, range 65‐78		
Schnutenhaus et al 2020	Prospective clinical study, RCT	AZ, PZ	SI	s‐CAIS	48/48	1	n/a	No significant differences in ISQ value between ARP and control groups. Mean ± SD		
									ARP	Not grafted
								B	63.00 ± 8.75	64.12 ± 7.88
								M	63.48 ± 9.60	65.15 ± 8.30
Smitkarn et al 2019	RCT	AZ, PZ	SI	s‐CAIS, FH	52/60	1.15	0.5 mo	Significant differences for both ISQ and ITV median (IQR)		
									s‐CAIS	FH
								ISQ at B	63.5 (12)	72 (9)
								ISQ at M	65.0 (12)	72 (11)
								ITV	22.5 (20)	35 (11)

Abbreviations: ARP, alveolar ridge preservation; AZ, esthetic zone; B, vestibulo‐oral direction; d‐CAIS, dynamic computer‐assisted implant surgery; DL, delayed loading; FE, fully edentulous; FH, freehand placement; IL, immediate loading; IQR, interquartile range; ISQ, implant stability quotient; ITV, insertion torque value; M, mesio‐distal direction; n/a, the data were not provided in the articles; PE, partially edentulous; PS, posterior; RCT, randomized clinical trial; RT, reverse torque value; s‐CAIS, static computer‐assisted implant surgery; SD, standard deviation; SI, single implants.

##### Intraoperative complications of a technical nature

Reported intraoperative complications of a technical nature on s‐CAIS include fracture of the surgical guide, an ill‐fitting guide, and anatomic limitations/deficient access (Table 2). None of the identified six studies^13^, ^14^, ^15^, ^16^, ^17^, ^26^ included a control group.

###### Fracture of the surgical guide

Nocini et al,^14^ in a retrospective study on s‐CAIS full arch placement, reported one surgical guide fracture (1.54% of patients) in the mandible, which was repaired during the operation.

###### Instability/misfit/inadequate stability of the surgical guide

The misfit or inadequate stability of the surgical guide is a more frequent complication, as reported in three studies. Mouhyi et al^13^ investigated surgical guide fit as a primary outcome by defining and testing specific parameters (adaptability, open spaces/gaps, interferences/defined points above the teeth). They found that 34 guides (85%) showed an excellent fit, four (10%) were acceptable (adaptation was possible in postprocessing, in the laboratory), and two (5%) were inadequate for use. The authors also pointed out that the two guides with inadequate fit and stability were made from resin. They recommended avoiding delays in the treatment when working with resin guides, as the dimensions of these guides might change with time. Likewise, Mangano et al^15^ assessed the s‐CAIS surgical guide fit, with 24 of them (85.71%) exhibiting an optimal fit and stability, three (10.71%) showing an optimal fit but only sufficient stability, and one (3.5%) with an inadequate fit and unsatisfactory stability. Only in the latter case, the surgeon elevated a full‐thickness flap and proceeded with conventional implant placement. The authors speculate that the reason for failure was the long time that elapsed between the manufacture of the template and the surgery in this case (over 1 month) and a possible consequent deformation of the resin template. Peñarrocha et al,^16^ in a case‐control study, reported a frequent need to extraorally adjust ill‐fitting surgical guides (11 out of 24 guides); all the surgeries were nevertheless possible after intraoperative adjustments. Aydemir and Arisan,^26^ in a split‐mouth RCT comparing d‐CAIS and freehand implant surgery, found unsatisfactory stability of the radiopaque stent used in the registration procedure in two out of 30 patients (6.67%) in the d‐CAIS group. The authors commended that intra‐oral adaptation of the radiopaque stent was challenging and the surgery was postponed for these two patients until the whole process was repeated.

###### Anatomic complications, bone dehiscence

Derksen et al^17^ reported a bone dehiscence after the placement of two implants (1.37%) with s‐CAIS in one patient. The dehiscence was managed with simultaneous bone augmentation. Similarly, Nocini et al^14^ also reported the position of two out of 342 implants (0.58%) in one out of 65 patients to have deviated from the plan, causing a minor buccal bone dehiscence, which did not affect the implant stability, osseointegration, or final restoration.

###### Limitation of access/anatomy variation

CAIS, either static or dynamic, requires specific instruments that might differ from conventional freehand surgery. Certain anatomic limitations, such as restricted mouth opening and location of the implant site, can impact the effectiveness of CAIS. Lin et al,^18^ in a prospective study with the BenQ AB s‐CAIS system, found it was not possible to place seven out of 43 implants (16.28%) at the molar area in five out of 21 patients with the fully guided s‐CAIS because of limited mouth opening. Derksen et al^17^ reported that it was not possible to use the drill guide in the far distal area (position 37) out of 145 implants (0.68%) in one out of 66 patients. The CAIS was aborted, and a bone level implant was placed with the conventional freehand technique, supplemented by a simultaneous bone augmentation procedure after a dehiscence was detected.

#### 2.3.2 Postoperative (healing) complications

Reported postoperative complications included excessive pain, swelling, inflammation/infection, and loss of implant/osseointegration failure (Table 2) in six studies.^10^, ^11^, ^13^, ^18^, ^22^, ^27^ Three studies^10^ reported excessive pain and/or swelling between day 2 and 1 month after s‐CAIS, with the percentage of affected patients varying between 1.6%^10^ and 5.26%.^13^ D’Haese et al^11^ reported the loss of one out of 78 implants (1.28%) shortly after insertion using s‐CAIS and immediate loading protocol because of abscess formation, possibly caused by remnants of impression material. Similarly, Zhao et al^27^ reported the loss of one implant placed and immediately loaded in a patient with a history of bite reconstruction of a deep overbite. Lerner et al^22^ reported that two implants (1.8%) had peri‐implant mucosal inflammation after 3 months in function. Di Giacomo et al^10^ also found that two patients (3.22%) developed slight gingival inflammation during the follow‐up period, which was resolved after professional prophylaxis and plaque control instruction.

#### 2.3.3 Short‐term clinical outcomes, biological and technical complications

##### Nonosseointegration

Five studies reported implants without osseointegration after completion of the healing period, all of which concerned s‐CAIS placement^13^, ^15^, ^18^, ^23^, ^28^ (Table 4). Failure of osseointegration in cases of conventional loading^18^, ^23^, ^28^ was reported in 0.69%‐3.23% of the implants placed, affecting 1.52%‐9.09% of patients. In the case of immediate loading,^13^, ^15^ failure of osseointegration was reported in 3.23%‐4.28% of the implants, occurring in 9.09%‐9.72% of patients.

**TABLE 4:** Studies included in the analysis for nonosseointegration and survivor rate

Author/year	Design	Location	Edentulism	Type of surgery	No. of patients/implants	No. implants/patient	Observation period	Non‐osseointrgation	Survival (implant level)
Cristache et al 2021	RCT	PS	SI, PE	s‐CAIS FH	49/111 66/145	2.26 2.19	24 mo	no	100%
Derksen et al 2019	Prospective cohort study	PS	PE	s‐CAIS	66/145	2.19	24 mo	1 implant (0.65%)	99.30%
D’Haese et al 2012	Prospective clinical trial	AZ, PS	PE	s‐CAIS	13/78	6.00	12 mo	n/a	98.71%
Di Giacomo et al 2012	Prospective study	AZ, PS	PE, FE	s‐CAIS	12/62	5.16	30 mo	n/a	98.33%
Ko et al 2021	RCT	AZ, PS	SI, PE	s‐CAIS	72/187	2.59	12 mo	8 implants (4.27%) in 7 patients	100% (delayed loading) 83.4% (immediate loading)
Kunavisarut et al 2021	RCT	PS	SI	s‐CAIS FH	20/20 20/20	1 1	7 d	no	100%
Kuo et al 2021	Case series	AZ	SI, PE	s‐CAIS	10/10	1	12 mo	no	100%
Lerner et al 2020	Retrospective study	AZ, PS	PE, FE	s‐CAIS	12/110	9.16	12 mo	2 implants (1.81%) in 1 patient	98.20%
Meloni et al 2013	Prospective case series	AZ, PS	FE	s‐CAIS	12/72	6	24 mo	no	100%
Meloni et al 2013	Prospective clinical study	AZ, PS,	FE	s‐CAIS	10/60	6	12 mo	no	100%
Naziri et al 2016	Prospective clinical study	AZ, PS	SI, PE	s‐CAIS	n/a	n/a	3 mo	no	100%
Nocini et al 2013	Retrospective study	AZ, PS	FE	s‐CAIS	65/342	5.26	12‐60 mo (mean 32.87)	7 implants (2.05%) in 6 patients	96.50%
Mangano et al 2018	Prospective study	AZ, PS,	SI, PE	s‐CAIS	19/36	1.89	12 mo	no	100%
Mouhyi et al 2019	Retrospective study	AZ, PS	SI, PE	s‐CAIS	38/110	2.89	12 mo	n/a	98.18%
Peñarrocha et al 2012	Case control study	n/a	SI, PE	s‐CAIS FH	12/19 12/22	1.58 1.83	3 mo	n/a	94.8%, 95.4%
Pozzi et al 2021	Prospective cohort study	AZ, PS	PE, FE	d‐CAIS	10/60	6	14‐18 mo (mean ± SD 16.2 ± 1.7)	no	100%
Skjerven et al 2019	Prospective cohort study	n/a	SI	s‐CAIS	20/27	1.35	n/a	no	100%
Søndergaard et al 2021	RCT	n/a	SI	s‐CAIS FH	13/14 12/12	1.07 1	2 mo	no	100%
Velasco‐Ortega et al 2021	Prospective clinical study	AZ	FE	s‐CAIS	14/28	2	12‐84 mo (mean ± SD 44.7 ± 31.4)	no	100%
Vinci et al 2020	Retrospective study	AZ, PS	FE	s‐CAIS	14/100	7.14	12 mo	no	100%
Zhao et al 2014	Prospective clinical study	AZ, PS	PE	s‐CAIS	11/31	2.81	n/a	1 implant (3.22%)	93.50%

Abbrevitions: AZ, esthetic zone; d‐CAIS, dynamic computer‐assisted implant surgery; FE, fully edentulous; FH, freehand placement; n/a, the data were not provided in the articles; PE, partially edentulous; PS, posterior; RCT, randomized clinical trial; s‐CAIS, static computer‐assisted implant surgery; SD, standard deviation; SI, single implants.

#### 2.3.4 Survival, medium‐/long‐term clinical outcomes, and biological and technical complications

##### Survival

Four comparative studies reported the implant survival rate between CAIS and FH (Table 4). Three of them demonstrated 100% implant survival for both protocols,^9^, ^28^, ^29^ however, two of them^9^, ^29^ had a very short observation period, and only one study had 2 years of follow‐up.^28^ Peñarrocha et al^16^ reported a similar survival rate for s‐CAIS (94.8%) and FH (95.4%) in a case‐control study, without any statistically significant difference.

Apart from the few comparative studies, 17 studies reported survival rates of implants placed with different static or d‐CAIS protocols. Fourteen of these studies had a follow‐up of at least 1 year, one was 3 months,^30^ while in the other two,^28^, ^31^ the observation period was not reported. Among these, eight studies reported 100% survival of the implants without complications.^15^, ^25^, ^30^, ^31^, ^32^, ^33^, ^34^, ^35^ Seven studies reported overall implant survival of 93.5%‐99.3%.^10^, ^11^, ^13^, ^14^, ^17^, ^22^, ^27^ One study^12^ reported a low survival rate when s‐CAIS was used for immediate loading (83.4%) as opposed to delayed loading (100%). With regard to implants placed with d‐CAIS, one study^24^ reported 100% survival after an average 1.5‐year follow‐up.

##### Peri‐implant tissue conditions and related outcomes: mucositis/peri‐implantitis

The diagnosis of peri‐implant mucositis/peri‐implantitis is reported in four studies, none of which, however, provided detailed case definitions (Table 5). At 1‐year follow‐up, the prevalence of peri‐implantitis for CAIS‐placed implants varied from 0%^36^ to 1.8%.^13^ In the only comparative RCT, Almahrous et al^36^ found no statistically significant difference at 1 year between implants placed with the conventional freehand technique, as opposed to short implants placed with s‐CAIS in posterior maxilla. Velasco‐Ortega et al,^37^ in a prospective cohort study, followed up 22 fully edentulous patients with 198 flapless mandibular implants with s‐CAIS and immediate loading for an average of 84.2 ± 4.9 months. They found 18 (9.3%) of the 193 remaining implants in 10 patients (45.4%) were associated with peri‐implantitis. Peri‐implantitis was statistically significantly more frequent in patients who smoked (66.6%). Finally, in another study of 14 fully edentulous patients/28 flapless implants with s‐CAIS (average follow‐up 44.7 ± 31.4 months), Velasco‐Ortega et al^34^ found four implants (14.3%) in two patients (14.3%) associated with peri‐implantitis.

**TABLE 5:** Studies included in the analysis of outcomes related to the condition of the peri‐implant tissue

Author/year	Design	Location	Edentulism	Type of surgery	No. of patients/implants	No. implants/patient	Observation Period	Peri‐implantitis (%implants/% patients)	Soft tissue outcomes	Marginal bone loss, mean ± SD (mm)	Keratinized mucosa	Studied outcomes
Almahrous et al 2020	RCT	PS	PE	s‐CAIS FH	27/75 29/69	2.57	12 mo	s‐CAIS: 0/0 FH: n/a/4	*BOP* s‐CAIS: 11.1% FH: 0% *Plaque* s‐CAIS: 14.8% FH: 12%	n/a	*Patients* s‐CAIS: 100% FH: 92%	No difference in prevalence of peri‐implantitis, clinical detection of the keratinized mucosa around implants, PI and BOP between s‐CAIS and FH.
Kuo et al 2021	Case Series	AZ	SI, PE	d‐CAIS	10/10	1	12 mo	n/a	n/a	−0.76 ± 0.15	n/a	The d‐CAIS for single implant placement in the esthetic zone demonstrated acceptable marginal bone level changes
Meloni et al 2013	Prospective study	AZ, PS	FE	s‐CAIS	12/72	6	24 mo	n/a	*BOP* 3.8% *PD* 2.75 mm	−1.35 ± 0.25	n/a	Immediate s‐CAIS into fresh extraction sockets of fully edentulous ridges resulted in acceptable outcomes of marginal bone loss and peri‐implant mucosal condition.
Peñarrocha et al 2012	Case control study	n/a	SI, PE	s‐CAIS FH	12/19 12/22	1.7	3 mo	n/a	n/a	n/a	s‐CAIS: 2.9 mm FH: 3.2 mm	The attached vestibular mucosa width was greater in FH group than that in s‐CAIS group, no statistical analysis was provided.
Polizzi et al 2013	Retrospective study	AZ, PS	PE, FE	s‐CAIS	27/160	5.92	61.3 mo	n/a	*Plaque*: Implants 15% Patients 11%	−1.39 ± 1.88	n/a	Mucosal conditions were reported as: ‐ 90% normal mucosa ‐ 7% mild inflammation; ‐ 1.75% moderate inflammation and BOP
Pozzi, et al 2021	Prospective study	AZ, PS	PE, FE	d‐CAIS	10/60	6	16 mo	n/a	*BOP* Implants 14.5% *Plaque* Implants 7.15%	−0.53 ± 0.28	n/a	The d‐CAIS in fully edentulous patients demonstrated good treatment outcomes in marginal bone changes and peri‐implant soft tissue conditions.
Velasco‐Ortega et al 2021	Prospective clinical study	AZ, PS	FE	s‐CAIS	22/198	9	84 mo	9.3%/45.4%	n/a	−1.44 ± 0.45	n/a	1. Peri‐implantitis and 2. Marginal bone loss was higher in edentulous patients with history of smoking
Velasco‐Ortega et al 2021	Prospective cohort study	AZ	FE	s‐CAIS	14/28	2	44.7 mo	14.3%/14.3%	n/a	1.25 ± 0.94	n/a	1. Peri‐implantitis was more frequent in ‐ patients with a history of smoking and periodontitis. 2. Marginal bone loss was higher in patients ‐ older than 70‐y‐old ‐ followed more than 5 y

Abbreviations: AZ, esthetic zone; BOP, bleeding on probing; d‐CAIS, dynamic computer‐assisted implant surgery; FE, fully edentulous; FH, freehand placement; n/a, the data were not provided in the articles; PD, pocket depth; PE, partially edentulous; PS, posterior; RCT, randomized clinical trial; s‐CAIS, static computer‐assisted implant surgery; SD, standard deviation; SI, single implants.

#### 2.3.5 Secondary clinical outcomes that can be related to complications and pathology

##### Plaque index, bleeding on probing, probing depth

Assessment of plaque, bleeding, and/or probing depths around implants placed conventionally or with CAIS are reported in four studies, one of which is comparative (Table 5). In the only comparative RCT, Almahrous et al^36^ found no statistically significant difference at 1‐year follow‐up in plaque, bleeding, and probing depths between implants placed with the conventional freehand technique and s‐CAIS. Meloni et al,^30^ in a prospective case series with 12 fully edentulous patients and 72 implants, found the average probing depth (PD) at 24 months to be 2.75 ± 0.40 mm, and the average BOP value to be 3.8% ± 1.8%. Polizzi and Cantoni,^38^ in a 5‐year retrospective study with 27 partially and fully edentulous patients/160 flapless implants, reported a cumulative plaque score of 15% at implant level (24 implants) and 11% at patient level. With regard to the gingival condition at site level, they reported 90% of sites as “normal gingiva”, 7% with “mild inflammation”, and 1.75% with moderate inflammation and BOP. Pozzi et al,^24^ in a prospective case series with 10 fully edentulous patients/60 implants placed with d‐CAIS and followed up for at least 1 year, found average plaque and bleeding scores to be 14.5% ± 8.18% and 7.15% ± 4.4%, respectively, at site level (Table 6).

##### Keratinized mucosa

The presence of keratinized mucosa at implants placed with s‐CAIS and conventional FH has been assessed in two comparative studies (Table 5). Peñarrocha et al^16^ found the mean attached vestibular peri‐implant mucosa width to be 2.9 (range 1‐4) mm in the s‐CAIS group (n = 12/19 implants) vs 3.2 (2‐5) mm in the conventional freehand group (n = 12/22 implants) 3 months after implant placement. No statistical analysis was attempted. On the contrary, Almahrous et al^36^ found that the number of patients who had keratinized mucosa was higher in CAIS (27 patients, 100.0% for s‐CAIS vs 23 patients, 92.0% for FH); however, this difference was not statistically significant. There seems to be selection bias with regard to reporting outcomes related to keratinized mucosa, as some authors have reported the absence of keratinized mucosa as a contraindication for flapless placement with CAIS,^39^ thus excluding such patients from respective groups.

##### Marginal bone loss

Changes in marginal bone level around implants placed conventionally or with CAIS have been reported in six studies (Table 5), none of which was comparative. Velasco‐Ortega et al^34^ reported mean MBL of 1.44 (±0.45) mm after a period of 84.2 ± 4.9 months. They found the bone loss statistically significantly higher in smokers (1.75 ± 0.33 vs 1.34 ± 0.39). Polizzi and Cantoni,^38^ based on 27 edentulous patients followed up for 4‐5 years, found MBL of 1.39 (±1.88) mm. Meloni et al^30^ found average MBL from baseline to 24 months to be 1.35 ± 0.25 mm. In a shorter study (average follow‐up: 44.7 ± 31.4 months) of 14 fully edentulous patients/28 flapless implants with s‐CAIS and restored with overdentures, Velasco‐Ortega et al^34^ found mean MBL of 1.25 ± 0.94 mm after 44.7 ± 31.4 months. The authors found statistically significant predictors of MBL to be an age older than 70 years (1.50 ± 0.80 vs 0.91 ± 0.88; *P* = .0077) and an observation period of more than 5 years (1.66 ± 0.58 vs 0.93 ± 0.81; *P* = .0001). Pozzi et al,^24^ in a prospective case series with 10 fully edentulous patients/60 implants placed with d‐CAIS, found an average MBL of −0.53 ± 0.28 mm after 16.2 ± 1.7 months. In a case series, Kuo et al^25^ assessed 10 immediate esthetic flapless implants placed through d‐CAIS. The cumulative mean MBL between implant placement and the 1‐year follow‐up was −0.76 ± 0.15 mm.

##### Esthetic outcomes

Esthetic outcomes were assessed in three studies, none of which was Table 6 comparative. Fürhauser et al,^6^ in a retrospective study, assessed clinical outcomes of single‐tooth implants for delayed replacement of upper incisors in 27 patients using s‐CAIS after a mean follow‐up of 2.3 years. The authors found that mean deviation between planned and actual implant position at the implant shoulder (0.84, SD 0.44 mm) and at the apex (1.16, SD 0.69 mm) statistically significantly correlated with the average pink esthetic score (PES), as implants with PESs of at least 10 showed statistically significantly less positional deviation (0.71 ± 0.46 mm) than implants with compromised esthetics (1.05 ± 0.32 mm). Direction of inaccuracy was not associated with any PES variables. The amount of deviation, by contrast, had a statistically significant impact (median PES: 9.5, interquartile range: 8‐11) compared with more accurate implant positions (mean PES: 13, interquartile range: 12‐13).

**TABLE 6:** Studies included in the analysis for esthetic outcomes

Author/year	Design	Location	Edentulism	Type of surgery	No. of patients/implants	No. implants/patient	Observation period	Instruments	Studied outcomes
Fürhauser et al/2015	Retrospective study	AZ	SI	s‐CAIS	27/27	1	27.6 mo	1. PES 2. Accuracy of placement	1. Higher deviation between planned and placed implant position correlated with lower PES.
Kuo et al/2021	Case Series	AZ	SI, PE	d‐CAIS	10/10	1	12 mo	1. PES/WES	Patients were satisfied with implant therapy’s function and esthetic outcome in the esthetic zone.
Lerner et al/2020	Retrospective study	AZ, PS	PE, FE	s‐CAIS	12/110	9.17	12 mo	1. Patient satisfaction questionnaire (a) Overall, how satisfied are you with the treatment received? (b) Are you satisfied with the function of your implant supported restorations? (c) Are you satisfied with the esthetics of your implant‐supported restorations? (d) Are you satisfied with the clean ability of your implant‐supported restorations?	1. Soft‐tissue was stable in all patients and showed satisfactory esthetic results. 2. Complete‐arch fixed reconstruction by means of guided surgery and immediate loading of implants placed in fresh extraction sockets resulted in stable tissue outcomes and esthetics

Abbreviations: AZ, esthetic zone; d‐CAIS, dynamic computer‐assisted implant surgery; FE, fully edentulous; PE, partially edentulous; PES, pink esthetic score; PS, posterior; s‐CAIS, static computer‐assisted implant surgery; SI, single implants; WES, white esthetic score.

Lerner et al^22^ studied the soft tissue stability 1‐year postimmediate restoration of fully edentulous patients (n = 12 patients/110 immediate flapless implants) through clinical photographs, focusing on the stability of the papillae and the soft tissue contours, using an index modified from Fürhauser et al.^6^ The authors found no difference in the soft tissue contours, as well as some evidence of “tissue maturing” and growth of interimplant “papillae”. Finally, Kuo et al^25^ assessed PES and pink esthetic/white esthetic scores (WESs) in a case series with 10 immediate esthetic flapless implants placed through d‐CAIS. The median values of total PES/WES, PES, and WES at 1‐year recall were 17 (range: 15‐19), 8 (7‐9), and 8 (7‐10), respectively, with a score of 12 set as the threshold for clinical acceptability of total PES/WES.

#### 2.3.6 Main conclusions concerning the impact of CAIS on the frequency/extent of complications and clinical outcomes

The available evidence failed to show differences in terms of intraoperative complications, clinical primary stability, immediate postsurgical healing, survival, and osseointegration of implants placed with CAIS or freehand protocols.

Moreover, the use of static or d‐CAIS does not appear to lead to any different outcomes with regard to the morphology or inflammatory status of the peri‐implant tissue, at least in the short to medium term.

### 2.4 PROs and PRE of CAIS compared with conventional placement

Ten studies assessed PROs or PRE as primary or secondary outcomes (Table 7). The studies utilized a wide diversity of instruments, from validated questionnaires such as the Modified Dental Anxiety Scale, OHiP 14,^9^ McGill pain and health‐related quality of life (HRQoL) questionnaires,^40^ established practices such as 1‐10 visual analog scale (VAS) scores of PRE (pain, swelling),^8^, ^15^, ^41^ to nonstandardized custom‐made questionnaires.^22^, ^28^ Noncomparative studies often reported high levels of “satisfaction” using descriptive questionnaires^19^, ^22^, ^42^; such conclusions cannot, however, be extrapolated beyond the limits of each individual study.

**TABLE 7:** Studies included in the analysis for patient‐reported outcomes/patient‐reported experience

Author/year	Design	Location	Edentulism	Type of surgery	No. of patients/implants	No. implants/patient	Observation period	Instruments	Main results
Almahrous et al/2020	RCT	PS	PE	s‐CAIS FH	27/75 29/69	2.48	12 mo	1. Pain (verbal rating scale) 2. Difficulty of treatment (4‐step Likert scale) 3. Overall satisfaction (4‐step Likert scale)	No difference in PROs between s‐CAIS and FH of short implants in posterior maxilla at placement and after 1 y
Cristache et al/2021	RCT	PS	SI, PE	s‐CAIS	49/111	2.26	12 mo	Patient satisfaction (custom questionnaire ‐ 3 item/0‐10 VAS)	Patients who underwent fully digital workflow of s‐CAIS reported significantly better experience with the dental implant insertion a minimum value of 6 was noticed for PDW and 9 for FDW.
Engkawong et al/2021	RCT	AZ, PS,	SI, PE	d‐CAIS s‐CAIS FH	28/64 30/61 30/54	2.03	14 d	1. Patient’s perceptions (5‐step Likert scale Yao et al) 2. Patient’s expectations (VAS 10 cm) 3. Healing Outcomes (VAS 10 cm)	No difference in PROs between s‐CAIS, d‐CAIS and FH. Preoperative expectations appeared similar among all 3 groups, as well as postsurgery PRE
Kunavisarut et al/2021	RCT	PS	SI	s‐CAIS FH	20/20 20/20	1	7 d	1. MDAS 2. Healing outcomes (VAS 10 cm) 3. Oral health‐related quality of life	No difference in PROs between s‐CAIS and FH for single‐tooth implant surgery in the posterior area.
Lerner et al/2020	Retrospective study	AZ,PS	PE, FE	s‐CAIS	12/110	9.17	12 mo	Patient’s satisfaction (custom questionnaire/5‐step Likert scale)	The great majority of patients reported high level of satisfaction.
Peñarrocha, et al/2012	Case control study	n/a	PE, SI	s‐CAIS FH	12/19 12/22	1.71	3 mo	Healing outcomes (VAS 10 cm)	No significant difference in postoperative PROs between s‐CAIS and FH was reported.
SanchoPuchades et al/2019	RCT	AZ, PS,	PE	s‐CAIS FH	47/(n/a) 26/(n/a)	n/a	7 d	1. Healing outcomes (VAS 10 cm) 2. Oral health‐related quality of life (custom questionnaire/100 mm VAS)	No difference in intraoperative or postoperative PROs between s‐CAIS and FH
Søndergaard, et al/2021	RCT	N/A	SI	s‐CAIS FH	13/14 12/12	1.04	n/a	1. Intraoperative Discomfort (3 items, 0‐10 VAS) 2. Operator (student) satisfaction (0‐10 VAS)	No significant difference was found in intraoperative discomfort between s‐CAIS and FH implant placement by senior dental students.
Vercruyssen et al/2016	RCT	AZ, PS	FE	s‐CAIS	15/90	6	10 d	1. McGill Pain Questionnaire (a) NWC‐T (b) PRI‐T (c) Healing outcomes (100 mm VAS) (d) level of swelling (100 mm VAS)	No difference in all PROs after s‐CAIS and immediate or delayed loading.
Youk et al/2014	Questionnaire Survey	AZ, PS,	n/a	s‐CAIS FH	37/(n/a) 90/(n/a)	n/a	n/a	1. Healing outcomes (custom questionnaire/VAS) 2. Patient satisfaction (custom questionnaire/VAS)	Patients who underwent computer‐guided surgery reported statistically significant lower degree of pain and higher satisfaction than those under conventional surgery (postop questionnaire survey)

Abbreviations: AZ, esthetic zone; d‐CAIS, dynamic computer‐assisted implant surgery; FDW, Fully Digital Workflow; FE, fully edentulous; FH, freehand placement; MDAS, Modified Dental Anxiety Scale; n/a, the data were not provided in the articles; NWC‐T, number of words chosen; PDW, Partially Digital Workflow; PE, partially edentulous; PRI‐T, pain rating index; PRE, patient‐reported experience; PRO, patient‐reported outcome; PS, posterior; RCT, randomized clinical trial; s‐CAIS, static computer‐assisted implant surgery; SI, single implants; VAS, visual analog scale.

Five RCTs^8^, ^9^, ^29^, ^36^, ^43^ with a total of 282 patients (conventional freehand, n = 117; CAIS, n = 166), one small case‐control study,^16^ and one cross‐sectional survey,^42^ have assessed different aspects of PROs and PRE between patients with s‐CAIS/d‐CAIS and conventional placement controls. Furthermore, one RCT compared PROs between partially and fully guided CAIS,^28^ and another between CAIS with immediate and delayed loading.^40^

#### 2.4.1 Preoperative assessment of perceptions and expectations

Sancho‐Puchades et al^43^ suggested some influence of a “novelty effect” of CAIS on patients' presurgical expectations, as they found that the majority of patients favored the CAIS approach even without having prior experience of implant surgery (CAIS preferred by 83% vs conventional by 6% of patients). However, when a more detailed assessment of presurgical expectations was conducted by Engkawong et al^8^ (ie, anticipated symptoms duration and intensity) and Kunavisarut et al^9^ (ie, Modified Dental Anxiety Score), no statistically significant differences were found between the expectations of patients scheduled for CAIS or conventional implant placement.

#### 2.4.2 Intraoperative patient‐reported experience

Two RCTs assessed pain during the intervention day. Almahrous et al^36^ used a verbal rating scale range of 1‐4, while Sancho‐Puchades et al^43^ assessed intraoperative pain and discomfort on VASs completed by patients immediately after the surgery. Neither study found any statistically significant difference in the intraoperative pain reported by the patients in the two groups. In addition, Sancho‐Puchades et al^43^ also assessed patients' perceived duration of the surgery, without, however, finding any statistically significant difference. Interestingly, patients in all groups had a rather accurate perception of the duration of the surgery, not statistically significantly different to the actual time recorded by the operators. Likewise, in Engkawong et al,^8^ the majority of patients (72%) found the duration of surgery acceptable, with no statistically significant differences noted between static and d‐CAIS and conventional placement. The same was true for Søndergaard et al,^29^ who assessed patients' intraoperative discomfort during fully and partial s‐CAIS by means of a questionnaire, without recording any statistically significant difference. Rather than the technology used, it was the length of the surgery that correlated well with higher intraoperative discomfort.^43^

#### 2.4.3 Postoperative healing patient‐reported outcomes/patient‐reported experience

**FIGURE 2:**
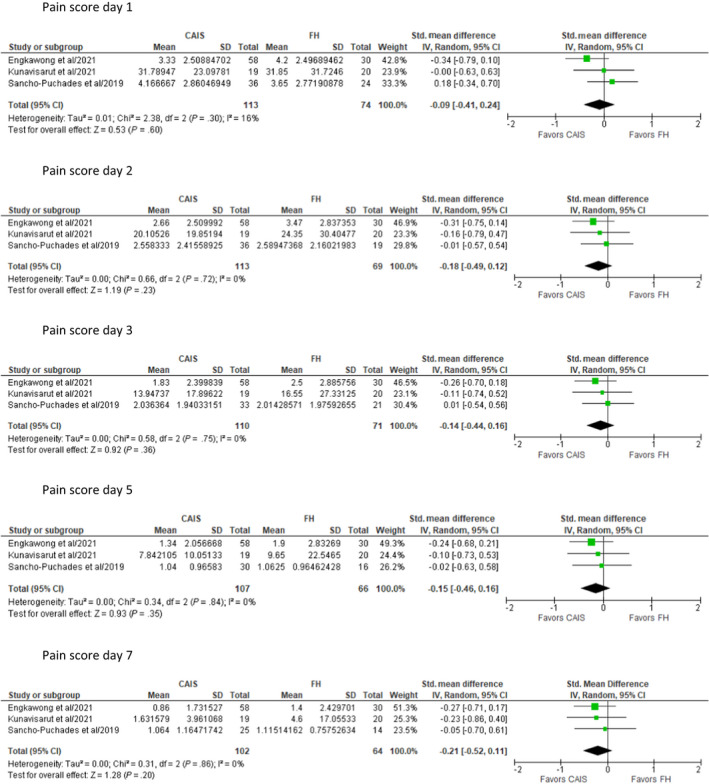
Meta analysis of three studies^9^, ^10^, ^43^ for daily postsurgical pain reported by patients after static and dynamic computer‐assisted implant surgery (CAIS) implant placement compared with those of freehand (FH) implant placement. SD, standard deviation

**FIGURE 3:**
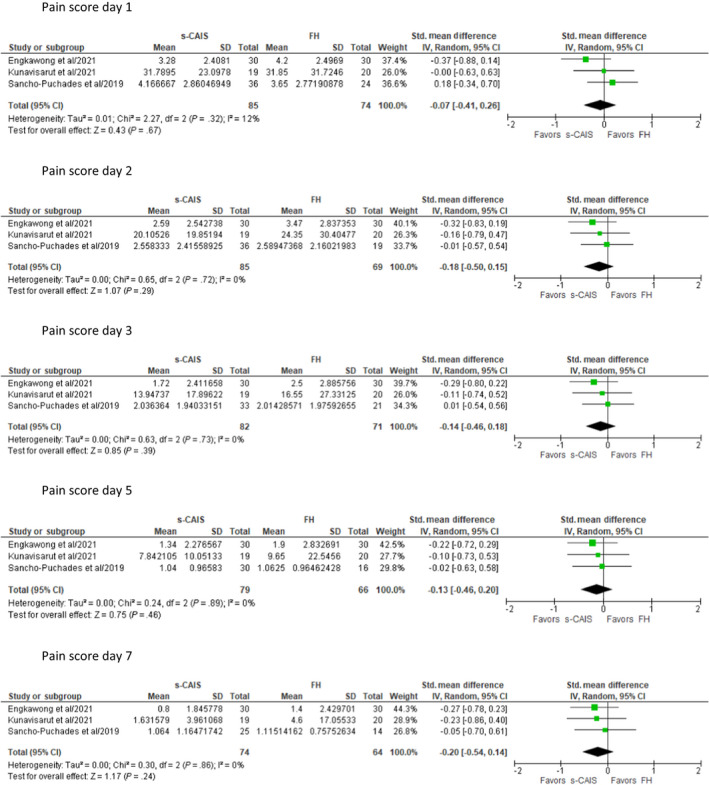
Meta analysis of three studies^9^, ^10^, ^43^ for daily postsurgical pain reported by patients after static computer‐assisted implant surgery (s‐CAIS) only, compared with those of freehand (FH) implant placement. SD, standard deviation

**FIGURE 4:**
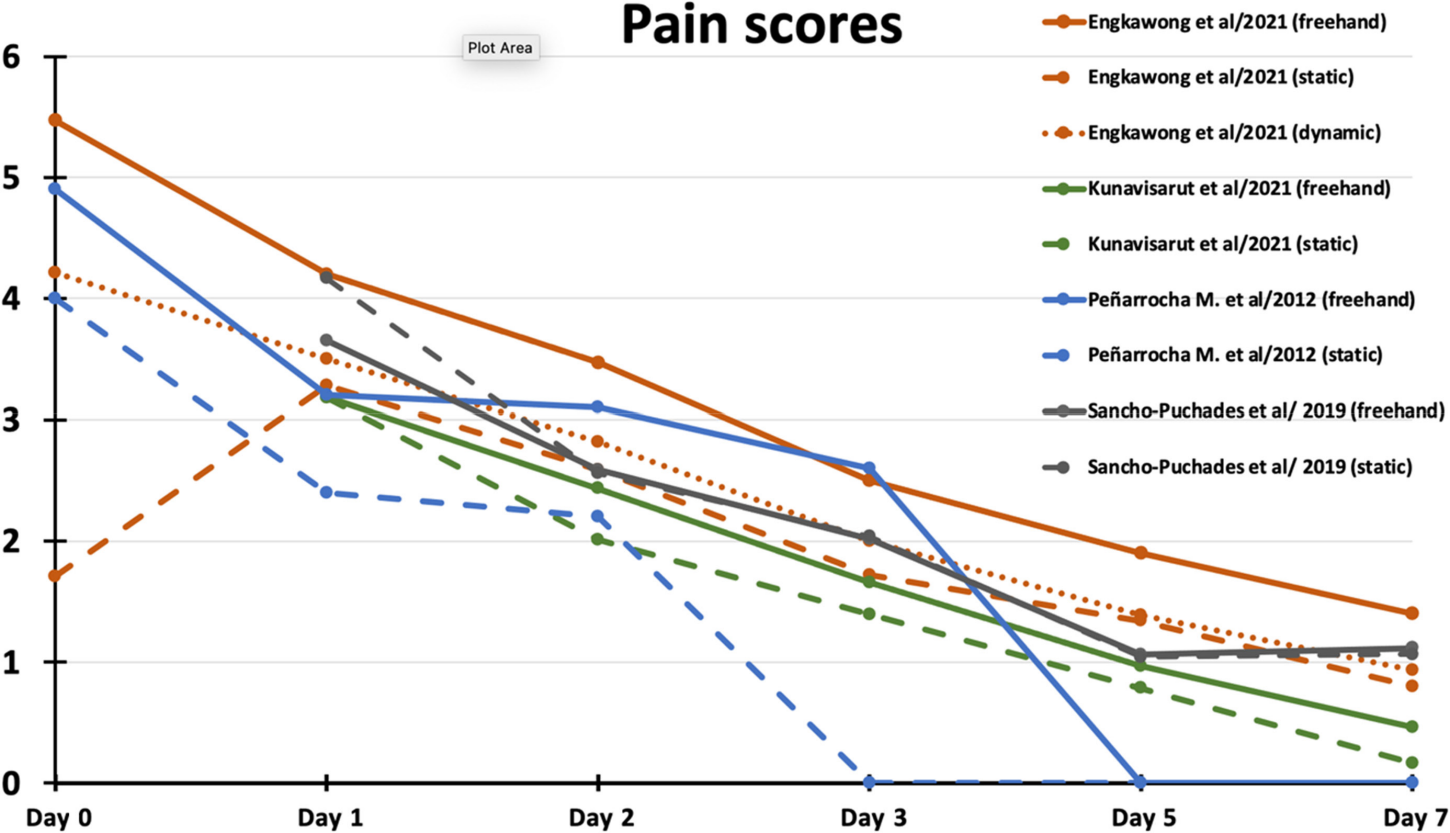
Average pain scores as recorded daily on visual analog scale 1‐10 by patients in four comparative studies^9^, ^10^, ^17^, ^43^

**FIGURE 5:**
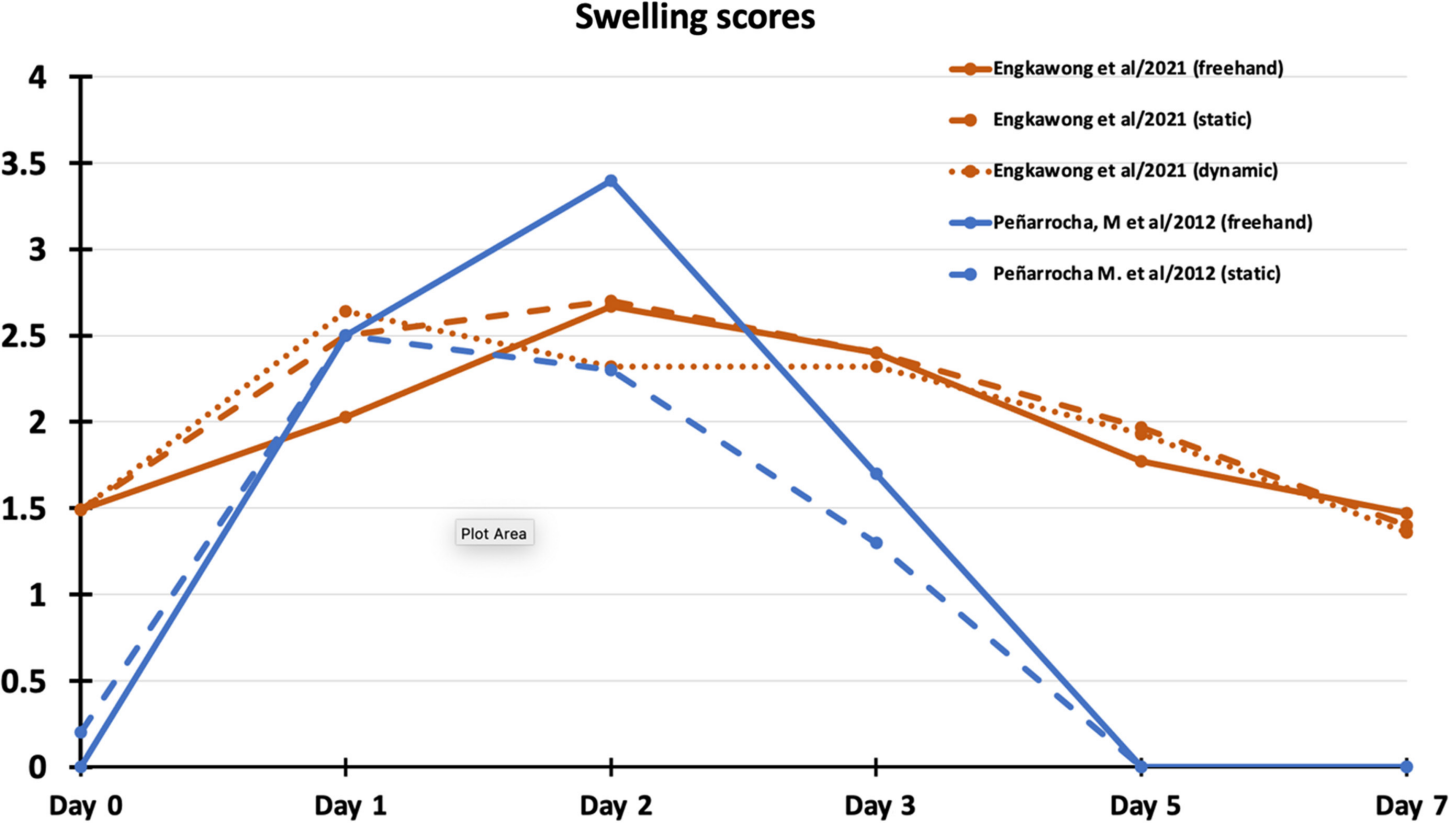
Average swelling scores as recorded daily on visual analog scale 1‐10 by patients in two comparative studies^9^, ^17^

Events associated with the immediate postsurgical healing are probably the most studied and reported patient‐reported outcome measures (PROMs), typically including symptoms observed in the first 7 days (pain, swelling, bruising, functional disturbances), as well as overall expressions of satisfaction. Five RCTs and one small case‐control study reported short‐term postoperative PROs/PRE adequately for collective analysis. Three RCTs^8^, ^9^, ^43^ utilized a VAS of 1‐10 to record pain and swelling among other outcomes but did not find any statistically significant difference in any major postoperative PROs. A small case‐control study (n = 24/41 implants) assessing the same outcomes did not attempt statistical analysis.^16^ The four above‐mentioned studies qualify for a meta‐analysis with regard to the outcomes of pain and swelling, thus the original raw data were requested from the respective authors. Data from three RCTs^8^, ^9^, ^43^ were made available and were included in the meta‐analysis conducted for pain and swelling during the first week after surgery (Figures 2 and 3). The meta‐analysis confirmed the trend favoring CAIS, which did not, however, reach statistical significant difference (Figures 2 and 3). All studies that followed daily outcomes using a VAS showed a similar pattern of reduction in the intensity of the symptoms (Figures 4 and 5). Pain reached a peak at 6 hours^8^, ^16^ or on the first day,^9^, ^43^ painkiller consumption on the first day,^8^, ^9^ and swelling on the second day after surgery.^8^, ^9^, ^16^, ^43^ It should be noted that all five previously mentioned studies utilized flap surgery together with CAIS. Vercruyssen et al^40^ utilized flapless CAIS comparing immediate with delayed full arch loading; however, the instrument used to record pain (MPQ‐DLV) was not directly comparable with the VAS 1‐10 scores. Nevertheless, the authors found no statistically significant differences regarding pain response treatment perception, or concerning the consumption or the type of painkiller.

#### 2.4.4 Postoperative oral health‐related quality of life and functional disturbance PROMs/patient‐reported experience

Kunavisarut et al^9^ assessed oral HRQoL by means of a 14‐item questionnaire covering oral function, daily activity, and postoperative symptoms for during the first 7 days after the surgery. Engkawong et al^8^ assessed six items related to postsurgery function disturbance, including the ability to perform oral hygiene, chewing, and speaking. Both of the above RCTs found no significant differences between s‐CAIS, d‐CAIS, and conventional surgery patients.

Vercruyssen et al^40^ found no statistically significant differences on HRQoL scores (a questionnaire consisting of 15 items assessing aspects of quality of life) when flapless CAIS was used for immediate or delayed full arch loading.

#### 2.4.5 Main conclusions concerning the impact of CAIS on patient‐reported outcomes and patient‐reported experience

Intraoperative and healing (pain, swelling, bruising, bleeding) events as well as postsurgery functional disturbances reported by patients did not differ statistically significantly between CAIS used with flap elevation and conventional implant placement.

### 2.5 Clinical outcomes related to the overall efficiency of CAIS compared with conventional placement

#### 2.5.1 Duration of the surgery with CAIS vs conventional implant placement

A widespread range of duration for surgical interventions has been reported by different authors, possibly reflecting the extent of surgery (eg, partial vs fully edentulous), the diversity of protocols and surgical setup, as well as defining different time points and measuring protocols (eg, continuous vs 5‐minute intervals). Comparisons of surgical duration are therefore only meaningful within studies with a comparative design, and eight studies were finally analyzed with regard to this outcome (Table 8). One RCT^8^ compared the surgical duration of static and d‐CAIS and conventional freehand, while three RCTs^36^, ^43^, ^44^ compared the duration of s‐CAIS and conventional freehand. Furthermore, one RCT compared the duration of d‐CAIS and conventional freehand surgery.^20^ Finally, one RCT,^28^ one prospective,^15^ and one retrospective study^13^ compared surgical duration between different protocols of s‐CAIS.

**TABLE 8:** Studies reporting duration of the surgery with computer‐assisted implant surgery vs conventional implant placement

Author/y	Design	Location	Edentulism	Type of surgery	No. of patients/implants	No. implants/patient	Observation period	Software	Studied outcomes
Almahrous et al/2020	RCT	PS	PE	s‐CAIS FH	27/75 29/69	3 2	12 mo	n/a	FH: 74.56 ± 31.66 min s‐CAIS (short implants): 72.79 ± 31.65 No significant difference was found
Engkawong et al/2021	RCT	AZ, PS	SI, PE	d‐CAIS s‐CAIS FH	28/64 30/61 30/54	2.28 2.03 1.8	14 d	1. IRIS‐100 (EPED Inc., Taiwan) 2. coDiagnostiX version 9 (Dental Wings, Canada)	1. FH: 70.3 min ± 47.08 was significantly shorter duration than s‐CAIS: 89.70 min ± 45.75. 2. d‐CAIS: 70.95 ± 42.48 took significantly shorter duration than s‐CAIS: 89.70 min ± 45.75. 3. No significant difference between d‐CAIS and FH.
Kaewsiri et al/2019	RCT	AZ,PS	SI	d‐CAIS s‐CAIS	30/30 30/30	1 1	12 mo	1. IRIS‐100 (EPED Inc., Taiwan) 2. coDiagnostiX version 9.7 (Dental Wings, Canada)	d‐CAIS: 15 min (12‐20 min) s‐CAIS: 18 min (13‐25 min) d‐CAIS + GBR: 40 min (30‐45 min) s‐CAIS + GBR: 48 min (30‐90 min). No significant difference between s‐CAIS and d‐CAIS was found.
Mangano et al/2018	Prospective clinical study	AZ, PS	SI, PE	s‐CAIS	19/36	1.89	12 mo	1. EXOCAD (Darmstad, Germany) 2. SMOP (Swissmeda, Switzerland)	Surgical guides with nonoptimal fit required 13.6 ± 1.5 min per implant as opposed to 11.4 ± 2.9 min per implant with optimal fit guides.
Mouhyi et al/2019	Retrospective study	AZ, PS	SI, PE	s‐CAIS	38/110	2.89	12 mo	SMOP (Swissmeda, Switzerland)	1. The mean duration was 23.7 ± 6.7 min per template. (median 22, 95% CI: 21.7‐25.7) 2. The mean duration was 6.5 min per implant.
Sancho‐Puchades et al/2019	RCT	AZ, PS	PE	s‐CAIS FH	47/n/a 26/n/a	n/a n/a	7 d	1. Simplant (Dentsply Sirona, USA) 2. SMOP (Swissmeda, Switzerland)	FH 92.88 min (± 39.8) s‐CAIS (a) 113.77 (±43.77) s‐CAIS (b) 142.77 (±47.25) No statistical analysis was conducted
Søndergaard et al 2021	RCT	PS	SI	s‐CAIS FH	13/14 12/12	1.07 1	n/a	MySimplant service (Dentsply Sirona, USA)	s‐CAIS: 70.65 min FH: 70.13 min No significant difference was found
Younes et al/2019	RCT	PS	PE	s‐CAIS FH	10/21 11/26	2.1 2.36	3 mo	Simplant 17.0 (Dentsply Sirona, USA)	s‐CAIS (fully): 40.10 min s‐CAIS (partial): 41.36 FH: 58.64 min s‐CAIS was significant faster than FH.

Abbreviations: AZ, esthetic zone; CI, confidence interval; d‐CAIS, dynamic computer‐assisted implant surgery; FH, freehand placement; GBR, Guided Bone Regeneration; n/a, the data were not provided in the articles; PE, partially edentulous; PS, posterior; RCT, randomized clinical trial; s‐CAIS, static computer‐assisted implant surgery; SI, single implants.

##### Static vs freehand: single implants and partially edentulous

Engkawong et al^8^ found a trend marginally above statistical significance (*P* = .07) for surgery duration to be shorter with conventional placement (70.3 ± 47.08 minutes) than with s‐CAIS (89.70 ± 45.75 minutes). A similar pattern was reported in the RCT conducted by Sancho‐Puchades et al^43^ on 73 partially edentulous patients, where the average duration for FH was 92.88 (± 39.8) minutes, while it was 113.77 (±43.77) and 142.77 (±47.25) minutes for two workflows of s‐CAIS, respectively, although no statistical analysis was attempted. Interestingly, the authors noted that an average of 6.91 and 5.56 minutes was invested for modifications of deficient fitting surgical guides in the two CAIS groups, respectively. The duration of implant placement reported by Almahrous et al^36^ was not statistically significantly different between FH (74.56 ± 31.66 minutes) and s‐CAIS of short implants (72.79 ± 31.65 minutes); however, it was noted that three implants were placed on average in the freehand group as opposed to two in the s‐CAIS group. Likewise, Søndergaard et al^29^ found no statistically significant difference in the duration of surgeries of posterior implants placed by senior dental students under flap with s‐CAIS or freehand. The mean time spent included discussion/instructions with supervisor and was 70.65 (41.25‐100) minutes for the CAIS group and 70.13 (55.5‐90) minutes for the freehand group. On the contrary, Younes et al^44^ found the mean duration of fully guided (40.10 minutes) and partially guided CAIS (41.36 minutes) to be statistically significantly shorter than that of freehand (58.64 minutes). However, the authors added that considerably more preoperative planning time was needed for guided surgery, resulting in the total time investment being similar for the CAIS and freehand groups. Mangano et al,^16^ in a study of flapless s‐CAIS, concluded that the average time required was slightly higher in the case of a nonoptimal template fit with 13.6 ± 1.5 minutes per implant as opposed to 11.4 ± 2.9 minutes when the surgical template did have an optimal fit.

##### Dynamic computer‐assisted implant surgery vs freehand and static computer‐assisted implant surgery: single implants and partially edentulous

Engkawong et al^8^ found d‐CAIS (70.95 ± 42.48 minutes) to be of similar duration to that of conventional placement (70.3 ± 47.08 minutes). The authors also noted a trend marginally above statistical significance (*P* = .07) for surgery duration to be shorter with dynamic than with s‐CAIS (89.70 ± 45.75 minutes). Likewise, Kaewsiri et al^20^ found no statistically significant difference in the duration of surgery between static and d‐CAIS, adding, however, an average of 3 (2‐5) minutes in d‐CAIS group due to the registration procedure. The average surgical time of static and d‐CAIS under flap in cases without bone augmentation was 15 (12‐20) and 18 (13‐25) minutes; in cases with Guided Bone Regeneration (GBR) it was 40 (30‐45) and 48 (30‐90) minutes. With a flapless approach, the average surgical time was 13 (12‐14) and 17 (12‐22) minutes for static and d‐CAIS, respectively.

#### 2.5.2 Influence of the experience and training of the operator

Outcomes related to the influence of the operator's experience and training have not been systematically assessed in comparative clinical trials. Relevant information can only be indirectly extracted from a small number of studies. Van de Wiele et al^45^ studied the outcomes of flapless s‐CAIS conducted by postgraduate students with those of experienced specialists from another study,^46^ where similar planning and clinical settings were utilized. The authors did not find any difference in the accuracy of placement between postgraduate students and specialists, but no PROs or other clinical outcomes were compared. Søndergaard et al^29^ conducted a clinical trial with implant placement by senior dental students utilizing s‐CAIS. Although the study suggested favorable outcomes with both fully and partial guided s‐CAIS, more interventions were required by the instructors to correct the angle of osteotomy in partial guided s‐CAIS, while some students working with fully guided s‐CAIS felt that the learning outcome was diminished, as they did not have to “think for themselves”. With regards to d‐CAIS, Block et al^47^ concluded that the highest accuracy was achieved after completing 20 patient cases, after studying the outcomes of consecutive surgeries by three implant surgeons. Some studies reported that right‐handed surgeons had lower accuracy when treating the left side of the patient compared with the right side.^45^, ^46^

#### 2.5.3 Main conclusions on length of surgery using computer‐**assisted implant surgery**

The evidence does not suggest any difference with regard to the length of surgeries with s‐CAIS on single implants or in partially edentulous patients.

However, differences between the evaluated CAIS systems and protocols, as well as in the way surgical time was calculated, can obstruct comparisons.

## 3 DISCUSSION

Although the application of CAIS has attracted a significant volume of research in the last 10 years, there is little evidence indicating superior clinical outcomes, both in terms of reducing complications and in improving patient experience. It is important to note, however, that the focus of research so far has been on assessing the accuracy of implant placement with CAIS, with most studies reporting little other than deviation from the planned implant position. Very few studies have systematically assessed clinical and PROs, with only a handful actually having primary outcomes other than accuracy. As the study of accuracy appears increasingly saturated, it is anticipated that the focus of research will gradually shift towards clinical and PROs, in particular with longer observation periods.

Another important characteristic of the currently available literature is that it is skewed towards the straightforward side of the clinical spectrum, with single gaps and partially edentulous patients being studied more, especially in the comparative segment of the literature. This might reduce the potential to document differences in clinical and PROs, which are indicated to be superior in the complex cases of fully edentulous patients and immediacy protocols. Again, the wider introduction of CAIS in clinical practice might expand the frontiers of research towards more complex protocols and patient scenaria.

Although no evidence of an impact of CAIS on the frequency and extent of complications was found, comparative studies are relatively few and the prevalence of intraoperative or early healing complications is in general already low at conventional placement. Also, the current consensus with the use of CAIS, which requires a “safety zone of 2 mm” from any sensitive anatomic structures,^1^ might hamper the ability to see a difference. The evidence suggests that the type of placement (eg, immediate vs delayed) might be a more important determinant for primary stability than the use of CAIS or not. With regard to the extent of mechanical stability, as this is measured by ITV, RFA, or RT, the evidence suggests that both static and d‐CAIS can reach the acceptable thresholds with high predictability. Whether FH or CAIS can reach higher values of ITV, RFA, or RT remains to be further investigated, as the available evidence is ambivalent.

Looking at medium‐ to long‐term outcomes, the use of CAIS does not appear to affect the morphology and condition of the peri‐implant tissue, as well as tissue inflammatory status. However, none of the comparative studies was longer than a year, a rather short period to assess certain outcomes, such as for example the occurrence of peri‐implantitis. Longer noncomparative studies on implants placed with CAIS have reported outcomes within the anticipated range of those with conventionally placed implants and demonstrating similar risk predictors (eg, smoking and years in function for MBL). Longer duration comparative studies utilizing accepted case definitions could clarify whether CAIS can lead to different long‐term tissue health outcomes. Assessment of clinical outcomes specific to esthetics has been scarce and not the subject of any comparative studies. Nevertheless, evidence from a retrospective study suggests a direct relationship between implant placement accuracy and esthetics measured by means of PES in the esthetic zone, while other smaller case series suggest PES/WES outcomes were above the acceptable threshold when CAIS was utilized.

**FIGURE 6:**
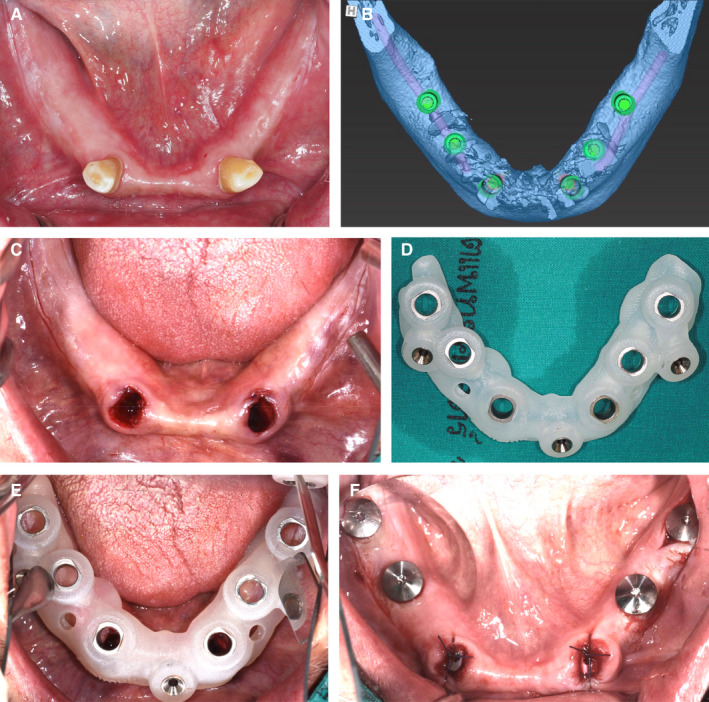
A, Partially edentulous mandible, with adequate bone volume and keratinized mucosa for full arch, implant‐supported, fixed dental prosthesis. B, Digital surgical plan for the placement of six implants with static computer‐assisted implant surgery (s‐CAIS) (the placement of six implants with 33, 43 immediate placement; 35, 37, 45, 47 with flapless placement). C, Extraction of teeth Nr. 33, 43. D, Fabrication of acrylic surgical guide (mucosa‐supported, acrylic with titanium sleeves, anchored with three fixation pins) for the s‐CAIS placement of two immediate and four flapless implants. E, Placement of s‐CAIS guide in the mandible. F, Final placement of six implants and healing abutments

The study of PROs and PRE has steadily increased in the last 10 years. Although patients appear to favor CAIS when asked prior to surgery, their actual expectations when it comes to specific aspects of the surgery, as well as presurgical stress levels, do not appear to differ statistically significantly. This might well be attributed to the well known “novelty effect”, which typically occurs with technologies the patients perceive as being innovative.^48^ Such a novelty effect might have a stronger influence when patients are asked to recollect experiences retrospectively at time points after treatment completion^49^ by means of treatment satisfaction questionnaires, as in Youk et al.^42^ At the same time, when more detailed outcomes measures were collected systematically during the respective phases of treatment, healing events, as well as postsurgery functional disturbances reported by patients, did not differ statistically significantly between CAIS used with flap elevation and conventional implant placement. Evidence points to the use of a flapless technique being a more potent determinant of postsurgical healing events. CAIS combined with flapless technique reduced the postsurgical pain statistically significantly and improved reported healing outcomes when compared with a similar CAIS protocol under flap.^39^ It is therefore reasonable to assume that the use of CAIS could indirectly contribute towards improving patient‐reported healing outcomes, to the extent that it can facilitate and empower wider use of flapless surgery (Figure 6).

**FIGURE 7:**
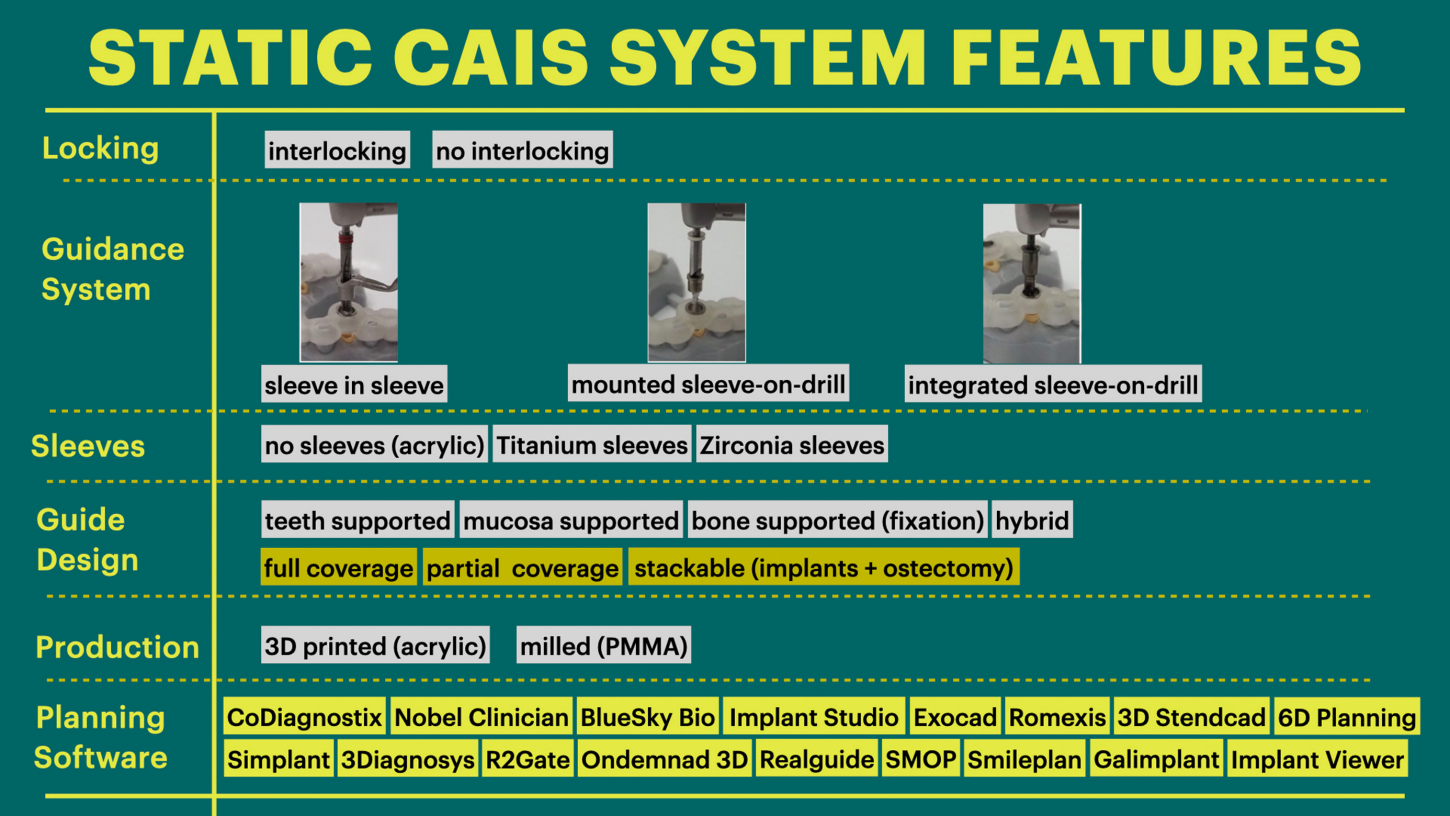
Overview of the principle characteristics of the static computer‐assisted implant surgery (CAIS) systems utilized in studies analyzed in this review. Implants were placed with diverse combinations of planning software, surgical guide design, and manufacturing techniques, as well as guided surgery drills and sequences. To this diversity one can add the potential influence of different hardware and algorithms of cone beam computed tomography, intra‐oral scanners, and hybrid digital‐analog protocols

Duration of the surgical intervention is an important parameter of success and efficacy, as well as patient experience, as an increased length of surgery is correlated with increased frequency and intensity of postsurgical pain and healing complications.^43^ Several authors have empirically reported CAIS to provide a “sensible reduction of surgery time”^14^, ^38^ in studies involving full arch rehabilitation; however, the data in the literature remain scarce and collective analysis is difficult. S‐CAIS is the best‐studied protocol with regard to the length of surgeries, and most studies on partially edentulous patients point towards no difference or even a possible trend for FH being faster. Even within the group of s‐CAIS, however, there is a wide diversity of protocols, software and hardware designs being utilized, each with the potential to impact clinical outcomes in different ways (Figure 7). In the case of the fully edentulous patients, past evidence^39^ showed no difference in surgery duration between conventional (68.71 ± 11.4 minutes) and s‐CAIS placement with a flap (60.94 ± 13.07 minutes), but found flapless s‐CAIS placement to be statistically significantly faster (23.53 ± 5.48 minutes). It is reasonable to expect that time reduction using CAIS will be more pronounced in multiple implant placement and may thus result in an overall shortening of treatment time in cases of higher complexity such as immediate loading of fully edentulous patients. It is also evident that factors such as raising a flap or not can impact the duration of surgery much more than the use of CAIS or not. The data are even more scarce in the case of the d‐CAIS, where only two RCTs were available. Although none showed statistically significant differences, they pointed to opposing trends, which can be attributed to the fact that one of the studies included the presurgery registration time in the overall calculated length of the surgery. This is indicative of the difficulty of synthesizing data from diverse protocols. In addition, different d‐CAIS systems have been shown to result in statistically significant differences in terms of surgery duration,^50^ possibly due to different technologies and specifications, while deficient fit of s‐CAIS guides is also known to increase the duration of the surgery. Conclusively, although there are limited data to suggest that s‐CAIS can substantially reduce the duration of implant placement surgeries for fully edentulous patients, the same remains questionable in the case of single implants and partially edentulous patients, where freehand surgery might at times be faster. To the extent that CAIS can increase the utilization of flapless surgery, its use could indirectly lead to a substantial reduction in time.

**FIGURE 8:**
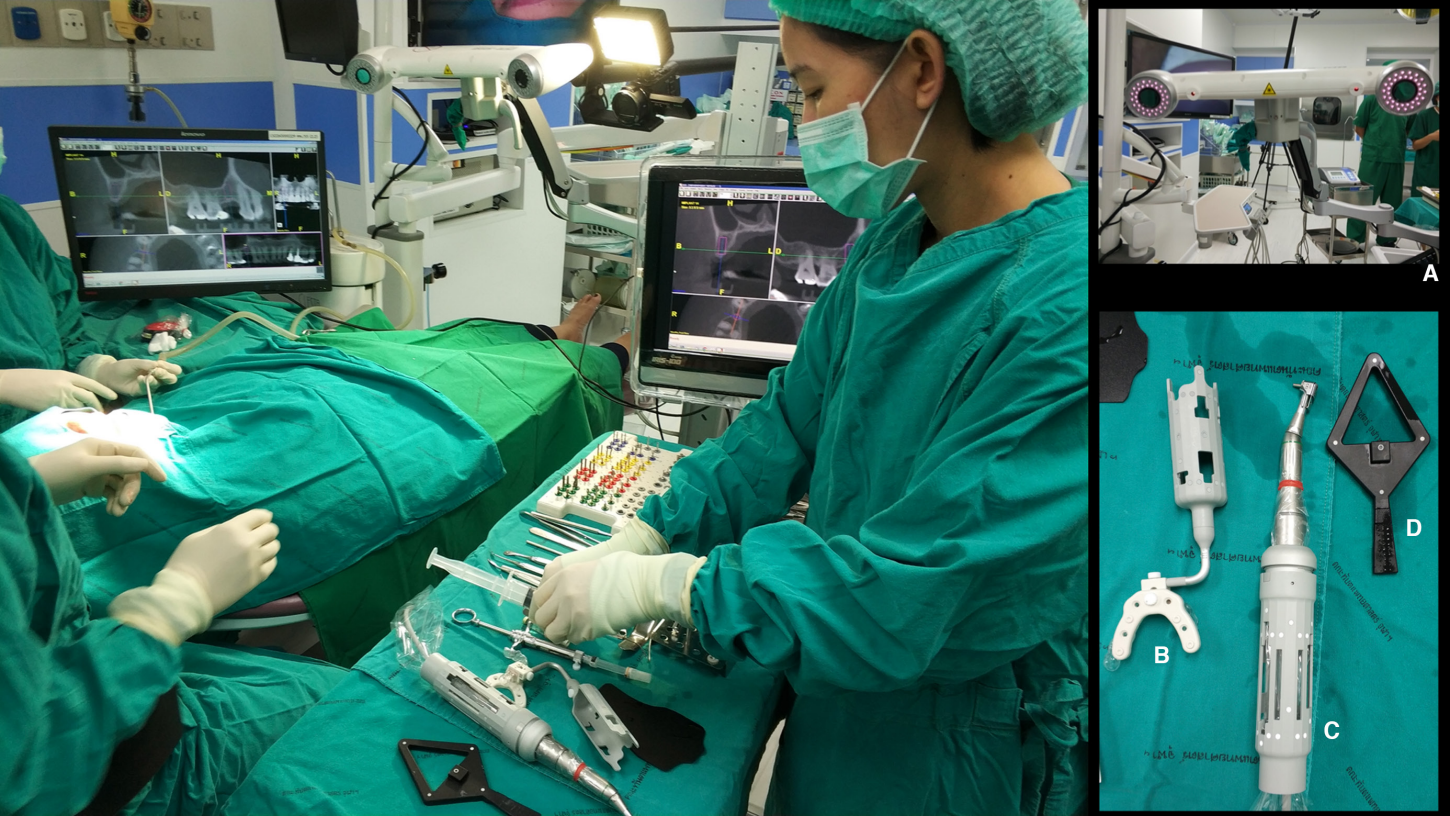
Dynamic computer‐assisted implant surgery systems employed in the studies analyzed in this review were first and second generation systems, typically based on: A, A set of optical tracking devices placed in the front or on top of the surgeon, B, Fiducial markers attached to a splint to be worn by the patient during the surgery, in order to register the jaw position in real time; C, Fiducial markers attached to the handpiece in order to register the position of the drill in real time; D, Calibration device to be utilized for alignment of the handpiece and the tracking device prior to the surgery. Third‐generation systems available today have further simplified some of these devices and procedures involved

At the same time, CAIS introduces a set of, mostly minor, technical problems and complications specific to each technology used. S‐CAIS is more frequently reported with technical problems, with the fracture of the surgical guide being the most severe, but very rare. Deficient fit and instability of the guide on the other hand are frequently reported, but only rarely led to abolishment of the CAIS protocol. Instead, intraoperative corrections have frequently taken place, which, however, can lead to a significant increase in the duration of the surgery.^15^ D‐CAIS has rarely been reported to have complications of a technical nature, but technical issues such as “loss of connection” between the sensors and the camera have been reported to be a nuisance, while system specifications, such as frame renewal rate, are indicated to significantly influence the duration of surgeries (Figure 8). Although not as wide as with s‐CAIS, high diversity exists within d‐CAIS software and hardware, which can potentially influence performance and clinical outcomes. At the same time, d‐CAIS is reported to be “operator sensitive” with a clinical study suggesting optimal results to be achieved only after treating at least 20 cases.^50^

The findings of this review should be seen under the limitations of the available literature, as well as those of the current review, which was limited to the last 10 years, as the evolution of protocols might hamper comparisons with outcomes from older studies. Nevertheless, some important older studies might still be relevant, and were therefore used in the discussion of the results where it was deemed appropriate.

## 4 SUMMARY AND CONCLUSIONS

1. The current evidence does not suggest any difference in terms of
  - intraoperative complications, immediate postsurgical healing and osseointegration, medium/long‐term survival and peri‐implantitis prevalence of implants placed with CAIS or freehand protocols;
  - intraoperative and early healing events as reported by patients (PROs and PRE) between CAIS with flap elevation and conventional implant placement;
  - length of surgeries in cases of single implants and partially edentulous patients, although there appears to be an advantage of CAIS for fully edentulous patients.
2. There is limited evidence that increased accuracy of placement with CAIS is correlated with superior esthetic outcomes.
3. Although CAIS as a sole parameter does not seem to consistently impact healing events, clinical and PROs, to the extent that it can increase the utilization of flapless surgery, predictability of immediacy protocols, and restorative‐driven implant placement, its use may indirectly lead to substantial improvements in all of the above parameters.
